# New approaches for metagenome assembly with short reads

**DOI:** 10.1093/bib/bbz020

**Published:** 2019-02-28

**Authors:** Martin Ayling, Matthew D Clark, Richard M Leggett

**Affiliations:** 1 Earlham Institute, Norwich Research Park, Norwich, UK; 2 Natural History Museum, London, UK

**Keywords:** Metagenomics, assembly, algorithms, sequencing

## Abstract

In recent years, the use of longer range read data combined with advances in assembly algorithms has stimulated big improvements in the contiguity and quality of genome assemblies. However, these advances have not directly transferred to metagenomic data sets, as assumptions made by the single genome assembly algorithms do not apply when assembling multiple genomes at varying levels of abundance. The development of dedicated assemblers for metagenomic data was a relatively late innovation and for many years, researchers had to make do using tools designed for single genomes. This has changed in the last few years and we have seen the emergence of a new type of tool built using different principles. In this review, we describe the challenges inherent in metagenomic assemblies and compare the different approaches taken by these novel assembly tools.

## Introduction

The increasing rate of metagenomic data generation represents a serious challenge to bioinformaticians tasked with analysing and understanding it. The EBI metagenomics portal alone has seen a tenfold increase in the number of processed samples from 2015–17, the last year a report was published [1] and as of January 2019, contains data for almost 140 000 samples and 1.9 million separate analyses. The emergence of next generation sequencing technologies brought with it the possibility to use shotgun sequencing to identify the composition of species within a heterogeneous sample. As high throughput sequencing technologies become more economical and widespread, the opportunity to sequence previously uncharacterised organisms directly from their environmental niche allows for a more complete view of the microbial world. Metagenomics, or envirogenomics, is a branch of genomics that seeks to explore the composition of complex communities of organisms. As many organisms cannot be grown in the laboratory (e.g. as many as 99% of bacteria are considered unculturable [2]), sequencing may be the only high throughput method to measure species diversity in many niches. Metagenomics has been applied to understanding a diverse range of environments including the human microbiome [3], the New York Subway [4], the virome of bats [5], the oceans [6, 7], the crop rhizosphere [8] and communities of extreme microbes living in geysers and hot springs [9]. Through metagenomic approaches, we can gain valuable insights into changing community profiles resulting from environmental changes. In traditional genomic studies, a single species is isolated and then cultured to produce a DNA-rich sample to assess. However, many species (particularly viruses) are impossible to study in this way and have thus remained unsequenced. Similarly, many studies have used 16S (prokaryotic) or 18S (eukaryotic) ribosomal RNA (rRNA) marker gene protocols, methods which, while simple and cheap, are unsuitable for detecting viral species and have limits in their discriminatory power for other classes of organism. By using whole-genome shotgun sequencing, it is possible to more completely explore environmental samples without prior isolation and culturing, enabling previously unsequenced species to be present in the resulting data sets. These opportunities pose new problems in data analysis, as metagenomic samples are inherently heterogeneous communities, sometimes containing tens of thousands of species [10, 11]. By considering a single bacterial genome in isolation, the problem of sequence assembly is relatively simple. However, the assumptions and simplifications that can be made in the case of a single genome are not applicable to heterogeneous environmental data sets. This presents both computational and conceptual obstacles necessitating the consideration of the more complicated problem of extricating as many genomes as possible from a complex mixture.

Though it is possible to analyse sequence data without assembly, most analyses can be improved by constructing longer more contiguous sequences (contigs). Next generation (Illumina) sequencing is comparatively cheap (~$12/Gb in July 2017 according to latest available NHGRI data at https://www.genome.gov/27541954/dna-sequencing-costs-data/), but the short read length limits the information within a single read. The identification of structures within a genome longer than a read, e.g. genes or operons, only becomes possible if reads are first assembled into longer sequence stretches. While metagenomic assembly is a research field still in its relative infancy compared to genomic assembly, the past few years have seen an increasing interest in its potential and the subsequent appearance of new software.

**Figure 1:**
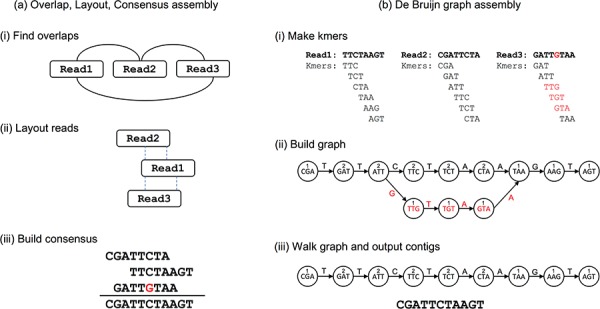
Two different approaches to genome assembly: (**a**) in Overlap, Layout, Consensus assembly, (i) overlaps are found between reads and an overlap graph constructed (edges indicate overlapping reads). (ii) Reads are laid out into contigs based on the overlaps (dashed lines indicate overlapping portions). (iii) The most likely sequence is chosen to construct consensus sequence. (**b**) In dBg assembly, (i) reads are decomposed into *k*mers by sliding a window of size *k* across the reads. (ii) The kmers become vertices in the dBg, with edges connecting overlapping kmers. Polymorphisms (red) form branches in the graph. A count is kept of how many times a kmer is seen, shown here as numbers above kmers. (iii) Contigs are built by walking the graph from edge nodes. A variety of heuristics handle branches in the graphs—for example, low coverage paths, as shown here, may be ignored.

In this review, we begin with a brief discussion of the genome assembly problem and then describe the specific challenges posed by metagenomic data. We describe the approaches taken by the main metagenomic assembly tools, drawing out common themes and identifying unique traits. We discuss approaches to simplifying huge data sets prior to assembly, as well as pipelines that contain assembly as just one step within a more involved analysis. Finally, we discuss what makes a ‘good’ assembly.

## (Single) genome assembly

Assembling short reads into contigs has many advantages. Longer stretches of sequence are more informative, allowing the researcher to consider whole genes or even gene clusters within a genome and to understand larger genetic variants and repeats. Additionally, it has the effect of removing most sequencing errors, though this can be at the expense of new assembly errors. First generation (Sanger) sequencing technology produced far fewer reads than second generation (or ‘next generation’) sequencing technology, but individual reads were longer (500–1000 bp). Assembly of Sanger data used overlap-layout consensus (OLC) approaches (Figure 1a), in which overlaps are computed by comparing all reads to all other reads, overlaps are grouped together to form contigs (layout) and finally a consensus contiguous sequence, or contig, is determined by picking the most likely nucleotides from the overlapping reads (e.g. Celera [12]). With the advent of second generation technologies, the number of reads increased exponentially, but the average length of a read shortened. This has enabled much reduced cost per gigabase of sequence, but the computational requirements of an OLC strategy become impractical due to the need to compare all reads with every other read in the data set (millions or even billions of reads).

To overcome this computational hurdle, de Bruijn graph-based assembly strategies were introduced [13] and have become widespread in the field. A de Bruijn graph (dBg) is a mathematical construct, where each vertex (or node) in a graph represents a *k*mer (a string of nucleotides of length *k*). Nodes in the graph are connected where the last k-1 nucleotides of one node overlap with the first k-1 nucleotides of another node. A graph is built by considering each read in turn, decomposing it into individual overlapping kmers and creating nodes for new kmers, updating coverage for existing kmers and adding vertices for new transitions (Figure 1b). In an ideal case, the dBg would form a single line, in which each node, apart from the two edges, is connected to one other node in the forward orientation and one in the reverse orientation. Converting such a graph into a hypothetical contig would be a trivial case of starting at one edge node and following labels to reach the second edge. Of course real data sets never result in such simple graphs and complex branching structures form as a result of errors, coverage differentials, heterozygosity, repeats and other structural variants. Thus much of the innovation in genome assembly algorithms has come from developing heuristics to simplify and navigate complex graphs consisting of millions of kmers to output contigs, as well as developing approaches to link contigs together in ‘scaffolds’. Some of these algorithms have stretched the strict mathematical definition of the dBg, but are nevertheless conceptually similar. The principle advantage of the dBg-based approaches is that, unlike OLC, there is no need to consider all input reads in a pairwise manner, making the problem instantly more tractable. Additionally, the repetition inherent in so many reads may be compressed, reducing memory requirements. The main drawback of the dBg is the loss of context that results from breaking reads into smaller kmers which can result in the joining up of disparate parts of a genome containing the same kmers—e.g. repeats. As a dBg becomes complex, ascertaining the most likely paths through it (the predicted genomic sequence) can be difficult.

Most short-read assemblers that have been produced in the past decade utilise dBgs for these reasons. These include popular genomic assemblers such as Velvet [14], ABySS [15] and SOAPdenovo [16]. There are limitations inherent in dBg assembly, however, particularity the initial choice of the size of kmer with which to build the graph. Choosing an inappropriate kmer size when building a graph may dramatically affect the quality of an assembly. Smaller kmers lead to more connected graphs; larger ones provide more specificity and fewer loops, but are more disconnected as the result of gaps or errors within the read data or lack of coverage of the genome. An additional constraint is that the kmer size cannot practically exceed two less than the read length in order to generate at least two edges. Some assemblers have begun to use a variety of kmer sizes during assembly to mitigate this issue. Iterative De Bruijn graph Assembler (IDBA) builds graphs iteratively, starting with a small k-mer size, and using the predicted contigs as hypothetical reads with which to build the next graph with a longer kmer size [17]. SPAdes [18] employs analogous techniques, moving from graphs built using smaller kmer sizes to maximise connectivity combined with larger kmer sizes for simplicity. RAMPART [19] runs a range of different assemblers with an option to produce multiple assemblies with different kmers, and a report summarising the statistics of each one. These procedures have become feasible as a result of increased computational power, but also as a result of increased sequence throughput.

## The challenge of metagenomic assembly

### Unknown abundance and diversity

In genomic assembly, there is an expectation that a sample contains a single species (apart from any contamination, which can be screened for prior to assembly) that allows assembly tools to make certain assumptions. The expected coverage of the target genome can be predicted from the total size of the data set (the reads and their length) and the estimated size of the genome. In turn, it is assumed that nodes or edges in a graph occurring with very low coverage compared to the expected coverage are likely the result of sequencing errors or low level contamination, and the graph is simplified considerably by removing such nodes or paths. Similarly, nodes with much higher than average coverage can be assumed to be part of repeat structures. The typical optimal sequence coverage for a single genome assembler is in the 20–200× range, with a common ‘sweet spot’ of ~50× [20]. However, in metagenomic data sets this assumption and simplifications cannot be made. Lower coverage nodes may originate from genomes with a lower abundance, not from errors, and so should not be discarded out of hand. Compounding this problem, the number of species within a sample, and the distribution of abundances of species is unknown. Abundance in heterogeneous samples often follows a power law [21], which means that many species will occur with similarly low abundances making the problem of distinguishing one from another problematic. The low coverage of most species means de novo assembly is unlikely unless the genome in question is relatively small, and instead we are reliant on reference genomes or gene prediction models/homology for evidence of the presence of species. It is imperative that a metagenomic assembly tool conserve as much of the less abundant species sequence as possible.

### Related species

In a genomic study, it may be assumed that all sequence reads derive from the same original genome. In metagenomic studies, this is emphatically not the case, with a potentially huge diversity of species to consider. However, while distinguishing divergent species is already a difficult problem, an even more challenging problem is that of identifying closely related species or even strains within species. Often in metagenomic samples, a number of sub-species or strains are present, and this is particularly evident with viral communities that typically contain an abundance of haplotypes. Related species or subspecies introduce extensive overlaps in a kmer set, and therefore create assembly graphs that are considerably more complex as multiple genomes occupy much of the same kmer space. Branches or loops between these homologous regions make traversing the graph more complex, and if either species occurs with a low abundance, then identifying the presence of separate species will be difficult and deconvolving the graph is extremely complex. Mistakes at this point can lead to chimeric contigs containing sequence from more than one (sub-)species and a failure to capture the true diversity of the sample.

### Memory and processing challenges

In common with genomic assemblers, metagenomic assemblers need to store each kmer present in the graph along with details of outgoing vertices and the coverage count of the kmer, sometimes separated by read set. Each assembler will also store additional attributes that are important for its specific algorithmic approach—such as flags or paired read information. This means that each kmer often requires 10 or 100 s of bytes to store and a full graph may contain 100 s of millions or even billions of kmers. Metagenomic assembly brings additional processing challenges when compared to genomic assembly of similar sized organisms. Obtaining sufficient sequence coverage depth of a complex and diverse metagenomic sample can result in many times the volume of data than for a single organism, but excessive coverage can also result in observing more sequencing errors in the higher abundance genomes. As a consequence, a dBg built to represent a metagenomic sample can require greater amounts of memory than one built to represent a similarly sized genomic sample and will be more fragmented making graph traversal more complicated. These challenges are mitigated directly by some assemblers (for example, MEGAHIT uses a succinct dBg to reduce storage requirements [22]), but can also be tackled using preprocessing methods before assembly, such as screening out likely host reads, subsampling and digital normalisation [23] to limit the effect of sequencing errors, or filtering/binning of reads and graph partitioning.

### Initial classification of reads

An initial classification of reads within a data set into likely taxonomic bins can be an effective first step in a metagenomic study. Removing reads from the data set that are readily identifiable as derived from an available reference genome can streamline the process of assembling complex samples. MEGAHIT [22] stratifies the data set into reads that are well supported by coverage, and those that are not; less well supported reads can still be included in an assembly as long as they extend well supported contigs. VICUNA [24] provides an optional step to filter out non-target reads via multiple sequence alignments (MSAs). MetaCRAM [25], a metagenomic pipeline tool, uses Kraken [26], a kmer-based taxonomic identification tool, to initially align reads to reference genomes and remove any known sequences from a data set prior to assembly.

### Graph partitioning

As metagenomic samples often contain multiple closely related species, the assembly graphs are often linked by shared sets of kmers, but this can also occur purely as a result of sequencing errors. This presents a challenge to assembler heuristics and can lead to the production of chimeric contigs, which contain a mixture of sequence from multiple genomes. This would be problematic for two reasons: firstly, the chimeric contig in question may imply detection of a new species, a real possibility given the nature of metagenomics; and secondly, it would also reduce the likelihood of the contig mapping to either of the genomes underlying the chimera, thus affecting their abundance levels or even obscuring their presence. As a consequence, it can be preferable to pre-partition the graph into sub-graphs, sacrificing contiguity for a more accurate community profile overall.

In practice, assemblers partition graphs by attempting to identify the nodes that join what appear to be distinct subgraphs and removing them from the data set. These nodes are often assessed as belonging in separate graphs as a result of differences in coverage, taking advantage of the notion that different species will be present in the original sample at different abundances. This difference in coverage is typically exploited on a localised basis to avoid the problems associated with the global coverage assumptions of genomic assemblers. IDBA-UD [27] and MEGAHIT both remove a node from within a graph if its coverage is significantly different to its neighbouring nodes. As a result of the iterative nature of both of these assemblers, any read that is not present in the graph as a result of this node removal will still be considered in the next round of graph building and so might be placed elsewhere in the graph (i.e. it is not discarded at this point). MetaVelvet considers the overall distribution of kmers within the data set, aiming to identify distinct peaks in a kmer distribution and using this as an indication of separate genomes with those approximate abundances. The graph is then split along these lines, maintaining local coverage within a subgraph. The critical joining node is not removed from the graph, but rather corresponding nodes are introduced into each subgraph to maintain connectivity.

Omega, although utilising an overlap-based approach rather than a dBg-based one, performs a similar step to graph partitioning [28]. During the process of building contigs, a contig will be split apart if the coverage of a constituent read is ‘significantly’ below that of the rest of that contigs coverage.

Ray Meta [29] and MetaVelvet-SL [30] do not split graphs strictly based on coverage. MetaVelvet-SL trains a support vector machine (SVM) to recognise probable chimeric nodes and remove them. Ray Meta operates a heuristic graph traversal procedure, which is based upon the minimum and peak coverages for each given seed path (similar to unitig) through a graph.

### Read pair information

Read pairs—both short-range paired-end and long-range mate pair—are invaluable in *de novo* assembly of single genomes, providing links between disconnected contigs, scaffolding contigs and spanning areas of repeats. However, the benefits in metagenomic assembly are less clear cut, with paired information often lending support to more than one route through the graph. Some assemblers still attempt the same scaffolding process used in genomic assembly, but others (MEGAHIT [22], Omega [28], PRICE [31], SPAdes [18], BIGMAC [32]) instead used paired reads to detect and resolve chimeric contigs produced from the misassembly of different genomes. Given the uneven coverage and low abundance of many of the species in most metagenomic samples, this produces more useful assemblies.

## Approaches taken by metagenomic assemblers

Though initially most researchers used the common genome assembly tools to assemble metagenomic sequence data, the last few years have seen the emergence of a series of dedicated metagenomic assemblers. Here, we summarise the approaches taken, starting with the smaller group of assemblers that we have loosely characterised as based on overlap strategies and then considering those utilising dBgs. Within the overlap category, we include both assemblers working on OLC strategies and those that do not follow the OLC paradigm but do rely on using read overlaps rather than decomposing reads into kmers. Table 1 summarises the tools discussed and provides a brief comparison of their features.

**Table 1:** Metagenomic assembly tools: key concepts and references to publications

**Tool**	**Method**	**Key concepts**	**Reference**
BBAP	OLC	Blast-based overlap assembly, with optional intermediary assembly stage.	Lin *et al*. 2017 [33]
Genovo	OLC	Generative probabilistic model; applies a series of hill-climbing steps iteratively until convergence; randomly (CRP prior) picks a contig to align read ‘i’ to breaks up chimeric contigs by taking the edge reads off of contigs every ~5 iterations.	Laserson *et al*. 2011 [34] Afiahayati *et al*. 2013 [35]
IDBA-UD	dBg	Build graph; remove dead ends (<2 k-1); merge bubbles; break graph on progressive (local) depth; error correction in reads (map reads to confident contigs; reads which match in all but a few bases can be ‘corrected’ to map perfectly); use mate pair info to build a ‘local’ assembly, avoid repeats and chimeras; hold trivial contigs, remove reads; make next graph; after k_max, partitions graph, clips tips, based on progressive (local) depth; Paired end reads requires long contigs to be effective.	Peng *et al*. 2012 [27]
IVA (iterative virus assembler)	OLC	Aimed at viruses. Greedy kmer-based extension. The most abundant kmer in the set is used as a seed, and this seed is grown out using a read that perfectly maps to it. A new kmer is drawn from the prefix of this read, which must be much more abundant than any other of the same size and occur more than 10 times in the data set.	Hunt *et al*. 2015 [36]
MAP	OLC	Reads are filtered before overlap (reduce pairwise alignments made), simple paths found first, mate pair support used to simplify paths, edges removed with contradictory/insufficient mate pair support.	Lai *et al*. 2012 [37]
MegaGTA	dBg	Guided assembly targeting specific genes. Employs HMM profile model, iterative kmers and succinct dBg.	Li *et al*. 2017 [38]
MEGAHIT	dBg	Solid kmers (occur more than a set threshold) and mercy kmers (remainder); mercy kmers that occur between two solid kmers in a read are kept; build a succinct dBG (dBG with Burrows-Wheeler Transform); remove tips, bubbles, progressively remove low local coverage edges; increasing kmer size, extract kmers from contigs and reads, build next graph.	Li *et al*. 2015 [22] Li *et al*. 2016 [39)]
MetaVelvet	dBg	dBG is first built with Velvet; population structure estimated from coverage of nodes (poisson distributions); dBg is partitioned into hypothetical subgraphs (possibly different species) using these peaks as a guide; only nodes from primary distribution are considered—chimeric and repeat contigs are identified and split by paired end info and coverage differences. Assembly produced for primary distribution; procedure repeated for next.	Namiki *et al*. 2012 [40]
MetaVelvet-SL	dBg	Similar to MetaVelvet, but the decision for identifying chimeric contigs is done using an SVM trained on (Paired ends, coverage, contig lengths) for each dinucleotide (AA, AT...GG); a training set is generated from a similar population, the SVM is trained on this, then passed over the dBg for decomposition.	Afiahayati *et al.* 2015 [30]
Omega	OLC	Read prefix/suffix (+/−) are stored in hashes; graph is built of V(r); simple paths (1 in, 1 out) are contracted, and transitive edges are reduced; tips removed (<10r) and bubbles are removed (hold edges with more r); minimum cost flow analysis for short (<1000 bp) contigs; Mate pair inserts are estimated from the assembly now, used to support contigs; scaffolding with long mate pair reads; remaining unresolved contigs are merged on similar coverage.	Haider *et al*. 2014 [28]
PRICE	Hybrid	Reads are ‘collapsed’ if identical, then if near identical; then (single strand) dbg used to assemble (essentially)—greedy walking, start at highest coverage; identical contigs collapsed, then near identical contigs (ungapped) and finally gapped.	Ruby *et al*. 2013 [31]
Ray Meta	dBg	Extension of Ray—no graph partitioning performed, doesn’t use a single peak for kmer coverage, min and peak coverage are specific for each seed path; heuristics-based graph traversal; graph is coloured according to an expected taxonomic profile.	Boisvert *et al*. 2012 [29]
SAVAGE	OLC	Aimed at viral quasi-species recovery. Strict overlap conditions reproduce quasi-species assembly with minimal misassemblies.	Baaijens *et al*. 2017 [41]
Snowball	Iterative joining	Guided assembly targeting specific genes. Overlapping paired-end read are merged, then assigned to profile domains. Consensus reads assembled for each domain by iterative joining.	Gregor *et al*. 2016 [42]
SPAdes and metaSPAdes	dBg	SPAdes started out as a tool aiming to resolve uneven coverage in single cell genome data; metaSPAdes builds specific metagenomic pipeline on top of SPAdes. Multiple kmer sizes of dBG, starting with lowest kmer size and adding hypothetical kmers of (pref smallest useful size) to connect graph.	Bankevich *et al*. 2012 [18] Nurk *et al*. 2017 [43]
VICUNA	Overlap	A min hash algorithm based on pairwise genetic distance threshold, inexact matching first (reads with similar or identical hash are merged) and then string matching of prefix/suffix of hashes is matched; (optional) target-like reads are kept first (similar reads binned, similarity of bin is used), everything else removed.	Yang *et al*. 2012 [24]
Xander	dBg	Guided assembly targeting specific genes. Employs HMM profile model.	Wang *et al*. 2015 [44]

### Assemblers adopting overlap strategies

Genovo [34] was one of the first metagenomic assemblers and is built using a generative probabilistic model that applies a series of hill-climbing steps iteratively. At each step, Genovo considers the position of every read and attempts to assign it to a new contig; upon finding a sufficiently good alignment it is added to that contig, otherwise a new contig is created. The assembly of chimeric contigs is prevented by removing the edge reads from all contigs every five iterations; should those reads have been correctly placed originally, they will be placed there again in the following steps. Genovo has been used in the reconstruction of bacterial and viral genomes from metagenomic samples [45, 46], and an extension to the assembler that made use of paired-end read information was released later [35].

MAP [37] uses paired-end information and specifically aims to break apart chimeric contigs in the assembly. Reads are filtered before the overlap stage to reduce the pairwise alignments required by the process, and simple paths joining reads are discovered first. Paired-end reads are then used to support and simplify paths, with edges removed that are insufficiently supported in the data set.

Omega [28] addresses the computational difficulties of OLC-based assembly with a hash function built of the prefix and suffix of each read in the data set which it uses to compute overlaps. A bi-directed graph [47, 48] is built up by matching reads to one another, and this is simplified by removing transitive edges (reads that are completely contained within a larger contiguous structure). Minimum cost flow analysis is performed on the basis of string copy number, to simplify the graph further, and long mate pair information is used to scaffold the contigs. There is no explicit stage for resolving chimeric contigs; it is assumed that the nature of an OLC approach will hinder their formation.

SAVAGE [41] is an overlap-based assembler of viral quasi-species, which reconstructs individual haplotypes in the final assembly by conservatively building overlap graphs (with strict minimal overlap length and of sequence similarity requirements). BBAP (the BLAST-based assembly pipeline [33]) creates a partial intermediary assembly that acts as a pseudo-reference for the remainder of the assembly process. In addition to working as *de novo* assemblers, both SAVAGE and BBAP have reference-guided options that we consider in Reference-guided assembly and contig binning section.

VICUNA is targeted at viral populations and uses neither a dBg nor a strict OLC approach. Rather, it clusters similar reads together first by generating a minimum hash value for each read [24]. This is a storage and compute efficient way to represent reads as those that are identical or similar will share the same hash value. These reads are then used to construct contigs, based on shared kmers, and reads that appear in multiple hashes enable contigs to be merged. This is not guaranteed to detect all good suffix/prefix matches; however, so a further seed-based extension is performed on the now greatly reduced data set. The authors propose this for populations of diverse but non-repetitive genomes, with high but variable coverage.

IVA [36] was developed for use with RNA virus populations. It performs greedy extensions within the data set, starting with the most abundant kmer. This kmer is used as a seed, and this seed is grown outwards using a read that perfectly maps to it. A new kmer is drawn from the prefix of this read, and this kmer must also be common to the whole set; it must be much more abundant than any other kmer of the same size, and occur more than 10 times in the data set. Though this assembler is not designed primarily as a metagenomic assembler, the authors assert that it is capable of performing well with samples of uneven coverage, a problem encountered when assembling environmental or heterogeneous samples. The software has been used in the assembly of viruses such as Zika virus and H1N1 influenza [49, 50].

Following a similar strategy to IVA, PRICE (paired-read iterative contig extension) also builds an assembly using greedy paired-end extension [31]. PRICE requires initial contigs to start the extension; these can be from another assembler or can be a subset of the input reads. PRICE then follows a number of iterations of a three-step cycle. First, input reads are mapped to the edges of existing contigs; second, overhanging pairs of mapped reads are assembled; third contigs are collapsed to reduce redundancy. During sequence collapse, a dBg is used to compute overlaps for sequences shorter than a user-specified length; as such PRICE represents a hybrid approach. PRICE has been used with Bunyavirus [51] and multiple water sample-based metagenomic studies [52].

### dBg-based assemblers

In general when assembling using dBg-based tools, an a priori decision must be made about the size of the kmer in the underlying graph. This decision can greatly affect the resulting assembly—if the kmer size is too large, the resulting graph structure may be too disconnected, but if kmer size is too small, the graph may become overly connected making it harder to navigate paths through it. The IDBA family of assemblers (e.g. IDBA-UD) attempts to solve this problem by iterating through increasing kmer sizes, pruning the graph and merging bubbles (loops) along the way. The graph is broken up at points of significantly differing coverage, with information from paired-end data included (although this is less informative in metagenomic rather than genomic cases). IDBA-UD has been used for assembly of a diverse range of bacterial and viral metagenomes (e.g. [53, 54]). More recently, MEGAHIT has used the process of increasing kmer size in assembly, but coupled it with succinct dBgs that are more efficient in computational terms. This assembler is several orders of magnitude faster and requires significantly less memory in the process of assembly and shows further performance increases when graphical processor unit accelerated. This attention to computational performance as well as to assembly completeness has made MEGAHIT one of the most popular and accurate of the current crop of metagenomic assemblers.

Velvet, a popular genome assembler, has received two updates aimed at metagenomic assembly in the form of MetaVelvet [40] and MetaVelvet-SL [30]. In MetaVelvet, a dBg graph is built using Velvet and the population structure is estimated from the coverage of nodes (modelled as Poisson distributions). The graph is then partitioned into subgraphs (each a hypothetical different species) using these coverage peaks as a guide. Chimeric and repeat contigs are identified and split using paired-end information and local differences in coverage. This assumes that genomes are distinct mostly on coverage information (which will be relative to abundance), which may not be the case with low abundance genomes that are more susceptible to stochastic noise. MetaVelvet-SL (SL for supervised learning) is an extension of MetaVelvet that improves upon the decision-making process for identifying chimeric contigs. An SVM is trained on multiple criteria (paired-end information, coverage, contig lengths) for each dinucleotide pairing (AA, AT...GG); a training set is generated from a similar population to the sample, the SVM is trained on this, and then passed over the sample graph for decomposition.

Ray is another commonly used genomic assembly to have received a metagenomic adaptation in the form of RayMeta [29]. This is an extension of Ray, where no graph partitioning is performed, but unlike Ray (where a single peak coverage is expected for the whole graph and kmers with a significantly lower coverage are excluded); in RayMeta a localised coverage distribution is generated for each seed path. These graphs are then walked using heuristic methods. A big emphasis for RayMeta is on computational efficiency and a lot of effort has been focused on scalability and distributability across standard clusters. This enables complex data sets to be processed across a networked cluster of low memory machines, avoiding the need for expensive, large memory architectures.

Although not specifically a metagenomic assembler, SPAdes [18] is aimed at genome assembly from single cell data, but its core assumptions of uneven coverage also make it suitable for metagenomic assembly. It builds multiple dBgs with differing kmer sizes and adds hypothetical kmers to ensure a connected graph. Chimeric contigs that are produced by these hypothetical kmers are then identified and split in a later stage. metaSPAdes [43] incorporates SPAdes into a metagenomic assembly pipeline and introduces new heuristics for differentiating intergenomic repeats between species.

## Assembly pipelines

Above, we described metaSPAdes as a pipeline for metagenomic assembly that incorporates SPAdes. A number of other software pipelines are available that combine read pre-processing, metagenomic assemblers and post-assembly analysis. Perhaps, the most comprehensive example is MetAMOS [55], which, at the time of writing, supports almost 20 genomic and metagenomic assemblers, along with a wide range of pre-processing, filtering, validation and annotation tools. Users can create workflows containing combinations of the tools that are suited to their data sets.

InteMAP [56] integrates output from two dBG assemblers (ABySS, IDBA-UD) and one OLC assembler (Celera) by separately merging low and high coverage contigs from pairs of assemblers. The authors of EnsembleAssembler also argue that merging the output from dBG and OLC assemblies can produce improved results [57]. MetaCRAM [25] is focussed on efficient storage via compression of metagenomic data sets. It taxonomically classifies reads and then assembles unclassified reads using IDBA-UD. Both the aligned reads and the unaligned read assemblies are then compressed for storage.

## Reference-guided assembly and contig binning

Thus far, we have focussed on *de novo* assembly tools. However, the use of reference-guided approaches has the potential to increase contiguity and accuracy but is likely only suited to those microbial communities that are best served by reference databases. MetaCompass [58] implements a pipeline that first maps reads against reference data sets, then generates reference-guided contigs, polishes them with Pilon [59] and finally combines unmapped reads with the polished contigs using MEGAHIT.

SAVAGE and BBAP, introduced above, both have reference-guided options. In the case of SAVAGE, references can be used at the point of building an overlap graph. In *de novo* mode, an FM-index is used as the mechanism for computing pair-wise overlaps between the entire read set. In reference mode, reads are aligned against a reference set and overlaps between reads inferred from alignments to the reference. A similar principle operates for BBAP: in *de novo* mode, a reduced set of high redundancy unique representative reads (HRURs) is BLASTed reciprocally to compute overlaps; in reference mode, the HRURs are BLASTed against a reference set.

A number of methods exist for gene guided assembly. These have the potential to improve assemblies in organisms for which references are not available, but their utility is limited for completely novel gene families. Xander targets specific protein-coding genes by combining a protein profile Hidden Markov Model (HMM) with a dBG to create a single combined weighted assembly graph [44]. MegaGTA was introduced to improve on the speed and accuracy of Xander [38]. While Xander employs a Bloom filter to conserve storage, MegaGTA opts for a succinct dBG. MetaGTA keeps a profile HMM for gene targeting but adds an iterative kmer approach along the same lines as that used in MEGAHIT. Snowball also uses profile HMMs, but adopts a different approach to assembly [42]. It requires overlapping paired-end reads as input, merges each pair and then assigns to gene domains using a profile HMM. Within each domain, consensus reads are assembled into contigs by a process of iterative joining.

The output of a metagenomic assembler is a set of contigs. A logical next analysis step is to group contigs into sets that represent species or strains. In some cases, it may be possible to carry this out by aligning contigs against reference data sets, but much effort has also been devoted to unsupervised clustering. Numerous approaches have been developed that rely on sequence composition (such as kmer content) in order to bin contigs [60, 61]. MetaBAT performs pairwise comparisons of contigs by calculating probabilistic distances based on tetranucleotide frequency and then uses a k-medoid clustering algorithm to bin contigs to genomes [62]. CONCOCT utilises Gaussian mixture models to cluster contigs based on both sequence composition and coverage across multiple samples [63]. DESMAN can take the output of CONCOCT or other binning tools and characterise to strain level [64]. COCACOLA, like CONCOCT, makes use of sequence composition and coverage, as well as utilising information from alignment to reference genomes and the linkage of contigs resulting from paired-end reads [65].

For viral samples, VirGenA provides an option for reference-based assembly that aims to separate samples into intraspecies groups [66]. Its approach is to iteratively map reads to either a user-specified viral reference sequence or a MSA of a set of references. From these mappings, an MSA of reads is built in order to construct a consensus sequence. VirGenA is not entirely reference guided and will utilise *de novo* strategies to attempt to close gaps.

## Assessing assembly quality

With an ever-increasing range of metagenomic assemblers available, how can researchers choose the tool for their application? A recent review article provides an in-depth discussion of approaches for the assessment of genomes assembled out of metagenomes [67]. The N50 is a common metric that is casually used to imply the quality of an assembly. If all contigs in an assembly are ordered by length, the N50 is the minimum length of contigs that contains 50% of the assembled bases. For example, an N50 of 10 000 bp means that 50% of the assembled bases are contained in contigs of at least 10000 bp. This statistic only indicates the contiguity of the assembled bases, is easy to manipulate (e.g. tools make different decisions on removal of small contigs which they consider noise or chaff), and gives no measure of assembly accuracy. A new assembler could generate long strings of random As, Cs, Gs and Ts and achieve high N50 but with no accuracy to the underlying genome, indeed the N50 could even be larger than the biological genome. Thus while it is the most used assembly statistic, it must be used cautiously and its significance understood. For example, well-established assembly tools designed for single genomes may produce assemblies of metagenomic data sets with high N50 values. However, this may have been achieved by removing kmers representing lower coverage species or collapsing inter-strain variation e.g. sacrificing complexity for contiguity.

A new metric, the U50, was recently proposed to overcome the limitations of the N50, particularly when assessing microbial or viral metagenomic assemblies [68]. The authors define the U50 as ‘the length of the smallest contig such that 50% of the sum of all unique, target-specific contigs is contained in contigs of size U50 or larger.’ It is calculated by aligning reads against a reference set and filtering out non-unique contigs. As such, it relies on the presence of a good quality reference data set, something that is not always available.

Assembly contiguity is important—after all, the whole point of assembling a metagenomic data set is to obtain longer sequences for downstream analysis. However, the ability to capture the metagenomic diversity of a sample—including the lower abundance species and strains—may be equally important. Thus there is a compromise between the desire for long contiguous sequence and the desire for an accurate representation of community composition, possibly down to the strain level. The aim of the project should lead to a choice of assembler and assembly parameters—particularly kmer size—that moves the emphasis one way or another.

A number of tools exist for assessing metagenome assembly quality. MetaQUAST [69] performs a BLAST search of contigs against a database of 16S rRNA genes and will automatically download the top 50 references. It then performs a reference-based quality assessment of contigs that align to these references. Such an approach is limited only to bacterial sequences. BUSCO (benchmarking universal single-copy orthologs) uses gene content to assess assembly quality and completeness [70]. It comes with a database of single-copy vertebrate, arthropod, metazoan, fungi and eukaryotic genes, as well as a smaller set of prokaryotic universal marker genes. CheckM also uses the presence of marker genes to assess assembly quality, but incorporates information about the position of a genome within a reference genome tree and collocation of genes in order to improve accuracy [71].

The Assembly Likelihood Evaluation framework (ALE) evaluates genomic and metagenomic assemblies with a reference-free approach that measures consistency between the input reads and output assembly sequence using read quality, read pair orientation, read pair insert length, sequencing coverage, read alignment and k-mer frequency [72]. ALE uses Bayesian statistics to define two probabilities: (i) a probability distribution describing the likelihood of an assembly without any read information and (ii) the probability of a given set of reads being generated from an assembly. These probabilities are combined in the ALE score, or the probability that the assembly is correct, which can be used to compare assemblies of the same genome. The probabilities are determined via alignments of the reads onto the assembly and calculation of placement sub-score (how well each read agrees with the assembly), insert sub score (how well paired-end read insert distribution agrees with expected distribution) and depth sub-score (evenness of coverage). The tool can also facilitate visualisations of alignments through a number of genome browsers, which can be a useful first stage *ad hoc* analysis of assembly quality.

In the field of single genome assembly, contests have been used in an attempt to compare the performance of different algorithms. In metagenomic assembly, the Critical Assessment of Metagenome Interpretation (CAMI) set out to develop an ‘independent, comprehensive and bias-free evaluation’ of both binning and assembly methods [73]. Its success relied on developers of tools and pipelines being willing to submit answers to a set of challenges and the organisers received six entries to the metagenomic assembly contest. Contestants were required to submit reproducible assemblies of three simulated metagenomic communities that were created from real sequencing data of newly sequenced viruses, bacteria and their plasmids. The results demonstrated substantial differences between the assemblies produced by the six teams—for example, total assembly size ranged from 12.32 Mb to 1.97 Gb for a data set with an expected assembly size of 2.80 Gb. Results also varied substantially according to the parameter settings chosen for each tool. Notably, assemblers using multiple kmers performed better than those using a single kmer size. All tools struggled with assembly of closely related genomes and the authors describe this as an ‘unsolved problem’. Overall, there were three assembly tools that performed better—MEGAHIT, Meraga (MEGAHIT combined with Meraculous [74]) and Minia [75]—but it’s not clear that this will necessarily be the case with all data sets or in the hands of all researchers.

## Conclusion

Assembling genomes out of heterogeneous samples is an extremely challenging problem and one that remains unsolved. The first specialised metagenomic assembly (Genovo) tool was released comparatively recently, in 2011, and the intervening years have seen the introduction of a wealth of new tools. Picking the right tool and then picking the right parameters for a specific data set are not straightforward tasks. Projects like the CAMI competition can contribute to the understanding of the strengths and weaknesses of different approaches, but researchers will benefit from trying a range of tools and parameters for their data. As such, there is really no substitute for dedicated post-assembly analysis using both automated tools such as MetaQUAST and manual analysis by the researchers themselves.

The focus of this article has been on assembly tools for short-read metagenomics, as Illumina remains the dominant platform for metagenomics [1] due to the lowest cost per Gbp of sequence and the need for high depth of sequencing of metagenomics samples. New library methods for Illumina sequencers, e.g. Illumina synthetic long reads [76], Dovetail in vitro HiC [77] and 10× Genomics microfluidics-created read clouds [78] allow more contiguous assemblies. However, they require longer DNA (10 kb for synthetic long reads and over 50 kb for Dovetail and 10× Genomics) that may be hard to extract from all samples, especially without introducing bias. *In vivo* HiC cross-links DNA within live cells, allowing scaffolding similar to Dovetail, but uniquely it also allows binning of chromosomes and plasmids in the same original cells [79]. The next few years may see the release of new assembly tools that are able to better utilise these new kinds of short-read data in order to significantly improve contiguity and accuracy of metagenomic assemblies in the same way that they have done for genomic assemblies.

Researchers are also increasingly attracted to long-read technologies, e.g. from established Pacific Biosciences [80] or the cheap, portable Oxford Nanopore Technologies MinION [81]. Both may simplify the need for assembly (with individual reads spanning multiple genes) or allow for generation of much longer contiguous sequence. Assembly of reads from these third generation platforms abandons dBg approaches and returns to the Overlap/Layout/Consensus models used in the earlier days of Sanger sequencing. As yet, there are no published tools dedicated solely to assembly of metagenomes from third generation platforms, but impressive results are possible using genome assembly tools such as Canu [82] or the very computationally efficient Minimap [83]. As the cost comes down and the accuracy and yields improve, these new technologies are likely to seem increasingly attractive platforms for metagenomic experiments and there will likely be new metagenomic assembly tools devoted to them.

## Notes

Key Points

- Assembling metagenomic data sets requires different algorithmic approaches to the assembly of single genomes.
- Unlike genomic samples, metagenomic samples contain many different genomes at wildly different levels of abundance. This means the assumptions made by most genome assembly tools break down.
- In the past few years, we have seen the appearance of new tools aimed specifically at metagenomic assembly of short-read data. This article provides a review of these tools and the algorithmic approaches they employ.
- There is no single tool that is best for all samples and for all scientific questions. Tools can often be differentiated by the tradeoffs they make between contiguity and the ability to capture species and strain diversity.

## Notes

**Martin Ayling** is a scientific computing specialist at the Earlham Institute whose previous postdoctoral position concerned analysis of metagenomic data sets and developing metagenomic assembly algorithms.

## Notes

**Matthew Clark** is a research leader in Life Sciences at the Natural History Museum. His research tests and develops new sequencing technologies to classify samples and to understand historic genetic change.

## Notes

**Richard Leggett** is a group leader at the Earlham Institute. His research is focused on developing computational methods and tools for enabling new technologies and for analysing metagenomic data.

## References

[1] Mitchell AL, Scheremetjew M, Denise H, et al. EBI Metagenomics in 2017: enriching the analysis of microbial communities, from sequence reads to assemblies. *Nucleic Acids Res* 2017;46(D1):D726–35. 10.1093/nar/gkx967 PMC5753268 29069476

[2] Ling LL, Schneider T, Peoples AJ, et al. A new antibiotic kills pathogens without detectable resistance. *Nature* 2015;517:455–9. 25561178 10.1038/nature14098 PMC7414797

[3] The Human Microbiome Project Consortium Structure, function and diversity of the healthy human microbiome. *Nature* 2012;486(7402):207–14. 22699609 10.1038/nature11234 PMC3564958

[4] Afshinnekoo E, Meydan C, Chowdhury S, et al. Geospatial resolution of human and bacterial diversity with city-scale metagenomics. *Cell Syst* 2015;29(1):72–87. 10.1016/j.cels.2015.01.001 PMC4651444 26594662

[5] Baker KS, Leggett RM, Bexfield NH, et al. Metagenomic study of the viruses of African straw-coloured fruit bats: detection of a chiropteran poxvirus and isolation of a novel adenovirus. *Virology* 2013;441(2):95–106. 23562481 10.1016/j.virol.2013.03.014 PMC3667569

[6] Venter JC, Remington K, Heidelberg JF, et al. Environmental genome shotgun sequencing of the Sargasso Sea. *Science* 2004;304(5667):66–74. 15001713 10.1126/science.1093857

[7] Sunagawa S, Coelho LP, Chaffron S, et al. Structure and function of the global ocean microbiome. *Science* 2015;348(6237):1261359. 25999513 10.1126/science.1261359

[8] Turner TR, Ramakrishnan K, Walshaw J, et al. Comparative metatranscriptomics reveals kingdom level changes in the rhizosphere microbiome of plants. *ISME J* 2013;7:2248–58. 23864127 10.1038/ismej.2013.119 PMC3834852

[9] Strazzulli A, Fusco S, Cobucci-Ponzano B, et al. Metagenomics of microbial and viral life in terrestrial geothermal environments. *Rev Environ Sci Bio* 2017;16(3):425–54.

[10] Daniel R The metagenomics of soil. *Nat Rev Microbiol* 2005;3:470–8. 15931165 10.1038/nrmicro1160

[11] Nesme J, Achouak W, Agathos SN, et al. Back to the future of soil metagenomics. *Front Microbiol* 2016;7:73, doi: 10.3389/fmicb.2016.00073. 26903960 PMC4748112

[12] Myers EW, Sutton GG, Delcher AL, et al. A whole-genome assembly of Drosophila. *Science* 2000;287(5461):2196–204. 10731133 10.1126/science.287.5461.2196

[13] Pevzner PA, Tang H, Waterman MS An Eulerian path approach to DNA fragment assembly. *Proc Natl Acad Sci U S A* 2001;98(17):9748–53. 11504945 10.1073/pnas.171285098 PMC55524

[14] Zerbino DR, Birney E Velvet: algorithms for de novo short read assembly using de Bruijn graphs. *Genome Res* 2008;18(5):821–9. 18349386 10.1101/gr.074492.107 PMC2336801

[15] Simpson JT, Wong K, Jackman SD, et al. ABySS: a parallel assembler for short read sequence data. *Genome Res* 2009;19(6):1117–23. 19251739 10.1101/gr.089532.108 PMC2694472

[16] Li R, Zhu H, Ruan J, et al. De novo assembly of human genomes with massively parallel short read sequencing. *Genome Res* 2010;20:265–72. 20019144 10.1101/gr.097261.109 PMC2813482

[17] Peng Y, Leung HCM, Yiu SM, et al. IDBA—a practical iterative de Bruijn graph de novo assembler In: Berger B (ed). *Research in Computational Molecular Biology. RECOMB 2010. Lecture Notes in Computer Science,* Vol. 6044. Berlin, Heidelberg: Springer, 2010.

[18] Bankevich A, Nurk S, Antipov D, et al. SPAdes: a new genome assembly algorithm and its applications to single-cell sequencing. *J Comput Biol* 2012;19(5):455–77. 22506599 10.1089/cmb.2012.0021 PMC3342519

[19] Mapleson D, Drou N, Swarbreck D RAMPART: a workflow management system for de novo genome assembly. *Bioinformatics* 2015;31(11):1824–6. 25637556 10.1093/bioinformatics/btv056 PMC4443680

[20] Desai A, Marwah VS, Yadav A, et al. Identification of optimum sequencing depth especially for de novo genome assembly of small genomes using next generation sequencing data. *PLOS One* 2013;8(4):e60204 doi: 10.1371/journal.pone.0060204. 23593174 PMC3625192

[21] Matthews TJ, Whittaker RJ On the species abundance distribution in applied ecology and biodiversity management. *J Appl Ecol* 2014;52(2):443–54.

[22] Li D, Liu CM, Luo R, et al. MEGAHIT: an ultra-fast single-node solution for large and complex metagenomics assembly via succinct de Bruijn graph. *Bioinformatics* 2015;31(10):1674–6. 25609793 10.1093/bioinformatics/btv033

[23] Howe AC, Jansson JK, Malfatti SA, et al. Tackling soil diversity with the assembly of large, complex metagenomes. *Proc Natl Acad Sci U S A* 2014;111(13):4904–9. 24632729 10.1073/pnas.1402564111 PMC3977251

[24] Yang X, Charlebois P, Gnerre S, et al. De novo assembly of highly diverse viral populations. *BMC Genomics* 2012;13:475. 22974120 10.1186/1471-2164-13-475 PMC3469330

[25] Kim M, Zhang X, Ligo JG, et al. MetaCRAM: an integrated pipeline for metagenomic taxonomy identification and compression. *BMC Bioinformatics* 2016;17:94. 26895947 10.1186/s12859-016-0932-x PMC4759986

[26] Wood DE, Salzberg SL Kraken: ultrafast metagenomic sequence classification using exact alignments. *Genome Biol* 2014;15:R46. 24580807 10.1186/gb-2014-15-3-r46 PMC4053813

[27] Peng Y, Leung HC, Yiu SM, et al. IDBA-UD: a de novo assembler for single-cell and metagenomic sequencing data with highly uneven depth. *Bioinformatics* 2012;28(11):1420–8. 22495754 10.1093/bioinformatics/bts174

[28] Haider B, Ahn TH, Bushnell B, et al. Omega: an overlap-graph de novo assembler for metagenomics. *Bioinformatics* 2014;30(19):2717–22. 24947750 10.1093/bioinformatics/btu395

[29] Boisvert B, Raymond F, Godzaridis E, et al. Ray Meta: scalable de novo metagenome assembly and profiling. *Genome Biol* 2012;13:R122. 23259615 10.1186/gb-2012-13-12-r122 PMC4056372

[30] Afiahayati SK, Sato K Sakakibara Y MetaVelvet-SL: an extension of the Velvet assembler to a de novo metagenomic assembler utilizing supervised learning. *DNA Res* 2015;22(1):69–77. 25431440 10.1093/dnares/dsu041 PMC4379979

[31] Ruby JG, Bellare P, Derisi JL PRICE: software for the targeted assembly of components of (Meta) genomic sequence data. *G3* 2013;3(5):865–80. 23550143 10.1534/g3.113.005967 PMC3656733

[32] Lam K, Hall R, Clum A, et al. BIGMAC : breaking inaccurate genomes and merging assembled contigs for long read metagenomic assembly. *BMC Bioinform* 2016;17:435. 10.1186/s12859-016-1288-y PMC5084376 27793084

[33] Lin Y, Hsieh C, Chen J, et al. De novo assembly of highly polymorphic metagenomic data using in situ generated reference sequences and a novel BLAST-based assembly pipeline. *BMC Genomics* 2017;18:223. 28446139 10.1186/s12859-017-1630-z PMC5406902

[34] Laserson J, Jojic V, Koller D Genovo: de novo assembly for metagenomes. *J Comput Biol* 2011;18(3):429–43. 21385045 10.1089/cmb.2010.0244

[35] Afiahayati SK, Sato K, Sakakibara Y An extended genovo metagenomic assembler by incorporating paired-end information. *PeerJ* 2013;1:e196. 24281688 10.7717/peerj.196 PMC3817583

[36] Hunt M, Gall A, Ong SH, et al. IVA: accurate de novo assembly of RNA virus genomes. *Bioinformatics* 2015;31(14):2374–6. 25725497 10.1093/bioinformatics/btv120 PMC4495290

[37] Lai B, Ding R, Li Y, et al. A de novo metagenomic assembly program for shotgun DNA reads. *Bioinformatics* 2012;28(11):1455–62. 22495746 10.1093/bioinformatics/bts162

[38] Li D, Huang Y, Luo R, et al. MegaGTA: a sensitive and accurate metagenomic gene-targeted assembler using iterative de Bruijn graphs. *BMC Bioinformatics* 2017;18(Suppl 12):408. 29072142 10.1186/s12859-017-1825-3 PMC5657035

[39] Li D, Luo R, Liu CM, et al. MEGAHIT v1.0: a fast and scalable metagenome assembler driven by advanced methodologies and community practices. *Methods* 2016;102:3–11. 27012178 10.1016/j.ymeth.2016.02.020

[40] Namiki T, Hachiya T, Tanaka H, et al. MetaVelvet: an extension of velvet assembler to de novo metagenome assembly from short sequence reads. *Nucleic Acids Res* 2012;40:e155. 22821567 10.1093/nar/gks678 PMC3488206

[41] Baaijens JA, El Aabidine AZ, Rivals E, et al. De novo assembly of viral quasispecies using overlap graphs. *Genome Res* 2017;27:835–48. 28396522 10.1101/gr.215038.116 PMC5411778

[42] Gregor I, Schönhuth A, McHardy A Snowball: strain aware gene assembly of metagenomes. *Bioinformatics* 2016;32(17):i649–57. 27587685 10.1093/bioinformatics/btw426

[43] Nurk S, Meleshko D, Korobeynikov A, et al. metaSPAdes: a new versatile metagenomic assembler. *Genome Res* 2017;27:824–34. 28298430 10.1101/gr.213959.116 PMC5411777

[44] Wang Q, Fish JA, Gilman M, et al. Xander: employing a novel method for efficient gene-targeted metagenomic assembly. *Microbiome* 2015;3:32. 26246894 10.1186/s40168-015-0093-6 PMC4526283

[45] Gupta A, Kumar S, Prasoodanan VPK, et al. Reconstruction of Bacterial and Viral Genomes from Multiple Metagenomes. *Front Microbiol* 2016;7:469. 27148174 10.3389/fmicb.2016.00469 PMC4828583

[46] Vázquez-Castellanos JF, García-López R, Pérez-Brocal V, et al. Comparison of different assembly and annotation tools on analysis of simulated viral metagenomic communities in the gut. *BMC Genomics* 2014;15:37. 24438450 10.1186/1471-2164-15-37 PMC3901335

[47] Medvedev P, Brudno M Maximum likelihood genome assembly. *J Comput Biol* 2009;16(8):1101–16. 19645596 10.1089/cmb.2009.0047 PMC3154397

[48] Myers EW The fragment assembly string graph. *Bioinformatics* 2015;21(suppl 2):ii79–ii85. 10.1093/bioinformatics/bti1114 16204131

[49] Lahon A, Arya RP, Kneubehl AR, et al. Characterization of a Zika Virus isolate from Colombia. *PLoS Negl Trop Dis* 2016;10(9):e0005019. 27654889 10.1371/journal.pntd.0005019 PMC5031432

[50] Watson SJ, Langat P, Reid SM, et al. Molecular epidemiology and evolution of influenza viruses circulating within European swine between 2009 and 2013. *J Virol* 2015;89(19):9920–31. 26202246 10.1128/JVI.00840-15 PMC4577897

[51] Chandler JA, Thongsripong P, Green A, et al. Metagenomic shotgun sequencing of a Bunyavirus in wild-caught Aedes aegypti from Thailand informs the evolutionary and genomic history of the Phleboviruses. *Virology* 2014;464:312–9. 25108381 10.1016/j.virol.2014.06.036 PMC4157124

[52] Ross DE, Gulliver D Reconstruction of a nearly complete pseudomonas draft genome sequence from a coalbed methane-produced water metagenome. *Genome Announc* 2016;4(5):e01024–e01016. 27795237 10.1128/genomeA.01024-16 PMC5054310

[53] Norman JM, Handley SA, Baldridge MT, et al. Disease-specific alterations in the enteric virome in inflammatory bowel disease. *Cell* 2015;160(3):447–60. 25619688 10.1016/j.cell.2015.01.002 PMC4312520

[54] Di Rienzi SC, Sharon I, Wrighton KC, et al. The human gut and groundwater harbor non-photosynthetic bacteria belonging to a new candidate phylum sibling to Cyanobacteria. *eLife* 2013;2:e01102. 24137540 10.7554/eLife.01102 PMC3787301

[55] Treangen TJ, Koren S, Sommer DD, et al. MetAMOS: a modular and open source metagenomic assembly and analysis pipeline. *Genome Biol* 2013;14:R2. 23320958 10.1186/gb-2013-14-1-r2 PMC4053804

[56] Lai B, Wang F, Wang X, et al. InteMAP: integrated metagenomic assembly pipeline for NGS short reads. *BMC Bioinformatics* 2015;16:244. 26250558 10.1186/s12859-015-0686-x PMC4545859

[57] Deng X, Naccache SN, Ng T, et al. An ensemble strategy that significantly improves de novo assembly of microbial genomes from metagenomic next-generation sequencing data. *Nucleic Acids Res* 2015;43(7):e46. 25586223 10.1093/nar/gkv002 PMC4402509

[58] Cepeda V, Liu B, Almeida M, et al. MetaCompass: Reference-guided Assembly of Metagenomes. 2017 bioRxiv. doi: 10.1101/212506. PMC12570322 41015028

[59] Walker BJ, Abeel T, Shea T, et al. Pilon: an integrated tool for comprehensive microbial variant detection and genome assembly improvement. *PLoS One* 2014;9:e112963. 25409509 10.1371/journal.pone.0112963 PMC4237348

[60] Chatterji S, Yamazaki I, Bai Z, et al. CompostBin: a DNA composition-based algorithm for binning environmental shotgun reads In: Vingron M, Wong L (eds). *Research in Computational Molecular Biology. RECOMB 2008. Lecture Notes in Computer Science,* Vol. 4955. Berlin, Heidelberg: Springer, 2008.

[61] Kislyuk A, Bhatnagar S, Dushoff J, et al. Unsupervised statistical clustering of environmental shotgun sequences. *BMC Bioinformatics* 2009;10:316. 19799776 10.1186/1471-2105-10-316 PMC2765972

[62] Kang DD, Froula J, Egan R, et al. MetaBAT, an efficient tool for accurately reconstructing single genomes from complex microbial communities. *PeerJ* 2015;3:e1165 doi: 10.7717/peerj.1165. 26336640 PMC4556158

[63] Alneberg J, Bjarnason BS, de Bruijn I, et al. Binning metagenomic contigs by coverage and composition. *Nat Methods* 2014;11:1144–6. 25218180 10.1038/nmeth.3103

[64] Quince C, Delmont TO, Raguideau S, et al. DESMAN: a new tool for de novo extraction of strains from metagenomes. *Genome Biol* 2017;18:181. 28934976 10.1186/s13059-017-1309-9 PMC5607848

[65] Lu YY, Chen T, Fuhrman JA, et al. COCACOLA: binning metagenomic contigs using sequence COmposition, read CoverAge, CO-alignment and paired-end read LinkAge. *Bioinformatics* 2016;33(6):791–8. 10.1093/bioinformatics/btw290 27256312

[66] Fedonin GG, Fantin YS, Favorov AV, et al. VirGenA: a reference-based assembler for variable viral genomes. *Brief Bioinform* 2017;20(1):15–25 bbx079. doi: 10.1093/bib/bbx079. PMC6488938 28968771

[67] Olson ND, Treangen TJ, Hill CM, et al. Metagenomic assembly through the lens of validation: recent advances in assessing and improving the quality of genomes assembled from metagenomes. *Brief Bioinform* 2017 10.1093/bib/bbx098. PMC6781575 28968737

[68] Castro CJ, Ng TFF U50: a new metric for measuring assembly output based on non-overlapping, target-specific contigs. *J Comput Biol* 2017;24(11):1071–80. 28418726 10.1089/cmb.2017.0013 PMC5783553

[69] Mikheenko A, Saveliev V, Gurevich A MetaQUAST: evaluation of metagenome assemblies. *Bioinformatics* 2016;32(7):1088–90. 26614127 10.1093/bioinformatics/btv697

[70] Simão FA, Waterhouse RM, Ioannidis P, et al. BUSCO: assessing genome assembly and annotation completeness with single-copy orthologs. *Bioinformatics* 2015;31(19):3210–2. 26059717 10.1093/bioinformatics/btv351

[71] Parks DH, Imelfort M, Skennerton CT, et al. CheckM: assessing the quality of microbial genomes recovered from isolates, single cells and metagenomes. *Genome Res* 2015;25:1043–55. 25977477 10.1101/gr.186072.114 PMC4484387

[72] Clark SC, Egan R, Frazier PI, et al. ALE: a generic assembly likelihood evaluation framework for assessing the accuracy of genome and metagenome assemblies. *Bioinformatics* 2013;29(4):435–43. 23303509 10.1093/bioinformatics/bts723

[73] Sczyrba A, Hofmann P, Belmann P, et al. Critical assessment of metagenome interpretation—a benchmark of metagenomics software. *Nat Methods* 2017;14:1063–71. 28967888 10.1038/nmeth.4458 PMC5903868

[74] Chapman J, Ho I, Sunkara S, et al. Meraculous: de novo genome assembly with short paired-end reads. *PLoS One* 2011;6:e23501. 21876754 10.1371/journal.pone.0023501 PMC3158087

[75] Chikhi R, Rizk G Space-efficient and exact de Bruijn graph representation based on a Bloom filter. *Algorithms Mol Biol* 2013;8:22. 24040893 10.1186/1748-7188-8-22 PMC3848682

[76] McCoy RC, Taylor RW, Blauwkamp TA, et al. Illumina TruSeq synthetic long-reads empower de novo assembly and resolve complex, highly-repetitive transposable elements. *PLOS One* 2014;27(5):757–67. 10.1371/journal.pone.0106689 PMC4154752 25188499

[77] Putnam NH, O’Connell BL, Stites JC, et al. Chromosome-scale shotgun assembly using an in vitro method for long-range linkage. *Genome Res* 2016;26(3):342–50. 26848124 10.1101/gr.193474.115 PMC4772016

[78] Weisenfeld NI, Kumar V, Shah P, et al. Direct determination of diploid genome sequences. *Genome Res* 2017;27(5):757–67. 28381613 10.1101/gr.214874.116 PMC5411770

[79] Stewart RD, Auffret MD, Warr A, et al. Assembly of 913 microbial genomes from metagenomic sequencing of the cow rumen. *Nat Commun* 2018;9:870. 29491419 10.1038/s41467-018-03317-6 PMC5830445

[80] Frank JA, Pan Y, Tooming-Klunderud A, et al. Improved metagenome assemblies and taxonomic binning using long-read circular consensus sequence data. *Sci Rep* 2016;6:25373. 27156482 10.1038/srep25373 PMC4860591

[81] Leggett RM, Clark MD A world of opportunities with nanopore sequencing. *J Exp Bot* 2017;68(20):5419–29. 28992056 10.1093/jxb/erx289

[82] Koren S, Walenz BP, Berlin K, et al. Canu: scalable and accurate long-read assembly via adaptive k-mer weighting and repeat separation. *Genome Res* 2017;27:722–36. 28298431 10.1101/gr.215087.116 PMC5411767

[83] Li H Minimap and miniasm: fast mapping and de novo assembly for noisy long sequences. *Bioinformatics* 2016;32(14):2103–10. 27153593 10.1093/bioinformatics/btw152 PMC4937194

